# Communications Between Bone Marrow Macrophages and Bone Cells in Bone Remodeling

**DOI:** 10.3389/fcell.2020.598263

**Published:** 2020-12-22

**Authors:** Kaixuan Chen, Yurui Jiao, Ling Liu, Mei Huang, Chen He, Wenzhen He, Jing Hou, Mi Yang, Xianghang Luo, Changjun Li

**Affiliations:** ^1^Department of Endocrinology, Endocrinology Research Center, The Xiangya Hospital of Central South University, Changsha, China; ^2^National Clinical Research Center for Geriatric Disorders, Xiangya Hospital, Changsha, China; ^3^Key Laboratory of Organ Injury, Aging and Regenerative Medicine of Hunan Province, Changsha, China

**Keywords:** macrophages, intracellular communication, macrophage-derived exosome, bone remodeling, bone diseases

## Abstract

The mammalian skeleton is a metabolically active organ that continuously undergoes bone remodeling, a process of tightly coupled bone resorption and formation throughout life. Recent studies have expanded our knowledge about the interactions between cells within bone marrow in bone remodeling. Macrophages resident in bone (BMMs) can regulate bone metabolism via secreting numbers of cytokines and exosomes. This review summarizes the current understanding of factors, exosomes, and hormones that involved in the communications between BMMs and other bone cells including mensenchymal stem cells, osteoblasts, osteocytes, and so on. We also discuss the role of BMMs and potential therapeutic approaches targeting BMMs in bone remodeling related diseases such as osteoporosis, osteoarthritis, rheumatoid arthritis, and osteosarcoma.

## Introduction

Macrophages are diverse, multifunctional, and plastic cells that regulate tissue homeostasis under physiological conditions and in various pathophysiological processes according to the surrounding environment.

Macrophages can be divided into circulating and resident macrophages. During embryonic development, macrophages are also the first emerged cells of the nascent immune system and they would infiltrate various developing organs to differentiate into tissue-resident macrophages, such as bone-resident macrophages (Wynn et al., 2013). Current knowledge of bone-resident macrophages (Michalski and McCauley, 2017) is limited. Osteal macrophages are a subset of bone-resident macrophages and are f4/80 positive and trap negative. Close to the bone surface, osteal macrophages are adjacent to osteoblasts, regulate bone formation, and are closely related to the osteogenic differentiation of mesenchymal stem cells. One characteristic of this group of myeloid cells is that although they share a common precursor with osteoclasts, they have different markers on their surfaces from osteoclasts. Communications between macrophages and other bone cells play an important role in bone tissue homeostasis and new bone formation. In this review, we focus primarily on the effects of macrophages on other bone cells. Extracellular vesicles are a group of cell-derived heterogeneous membranous structures that facilitate cell-cell communications. Hence, we discussed the potential contribution of the new-found microRNAs and alarmins contained in macrophage-derived extracellular vesicles in maintaining bone homeostasis in the context of bone stromal regulation. In the end, we further explained the role of macrophages in bone remodeling-related bone diseases and described the relationship between macrophages and bone tumors such as osteosarcoma.

This review focuses on the presence of macrophages in endosseous tissue, revealing the important role of macrophages in bone physiology and pathology.

## Macrophages

### The Occurrence and Function of Macrophages

Since Elie Metchnikoff first translated macrophages into Greek “big eaters,” macrophages are primarily known for their phagocytosis in inflammation and immunity (Gordon, 2008). Macrophages are differentiated immune cells with heterogeneity and plasticity. They are activated under different environmental signals and participate in diverse functions (Das et al., 2014, 2015; Jafarnezhad-Ansariha et al., 2018). Macrophage is often referred to as polarization. Different subtypes of macrophages have been derived from the simple M1/M2 classification, according to the environment, transcription factors, and cytokines secreted by macrophages. According to the expression of marker in macrophage surface, M1/M2 are currently also called “M1-like” and “M2-like” (Biswas and Mantovani, 2010), but in this review, we still only use M1 and M2 to represent.

**Figure 1:**
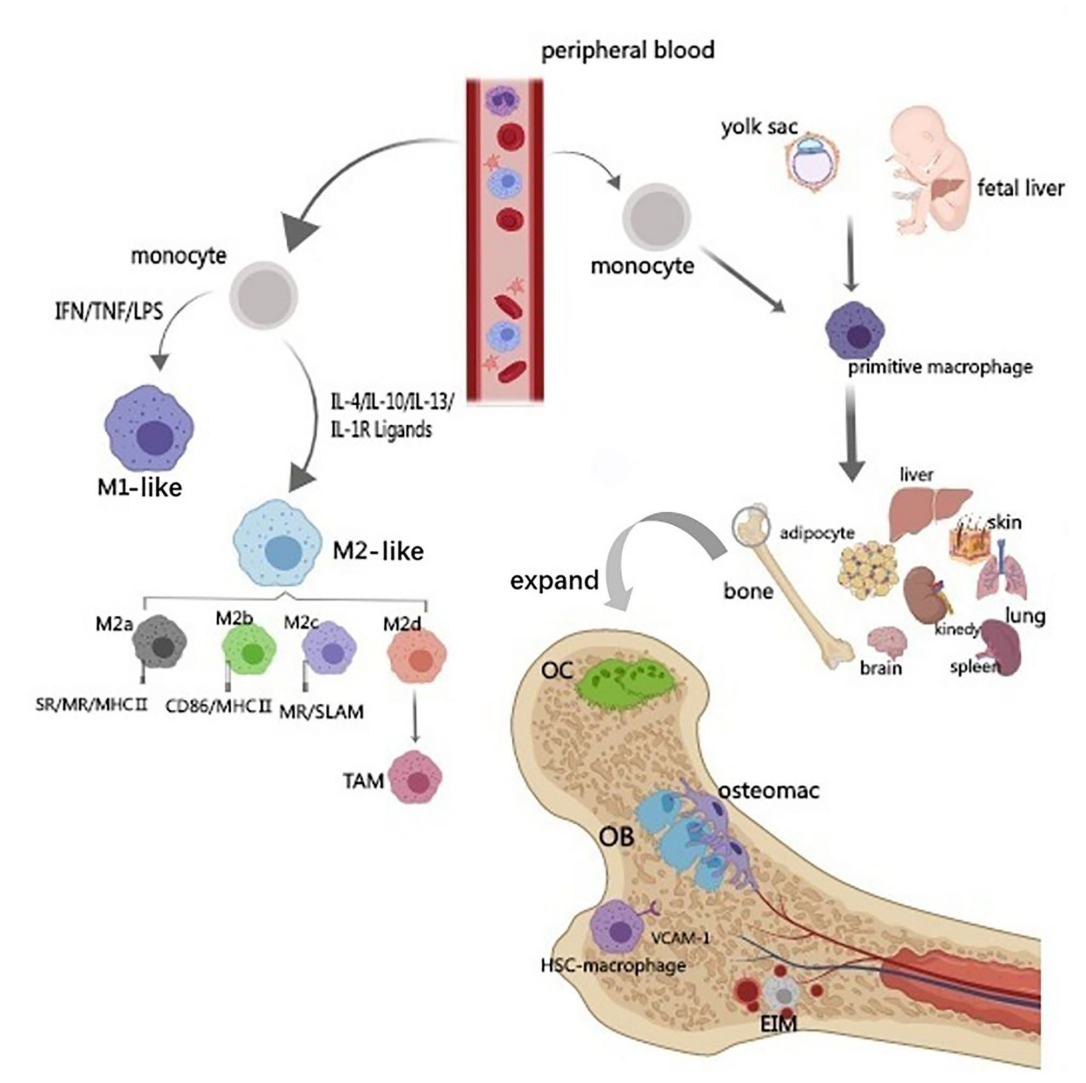
Origin and classification of macrophages. The diagram illustrates the source and classification of macrophages. Circulating macrophages are classified into M1 and M2 types according to their activators and functions. M2-type can be further subdivided into M2a, M2c, and M2d by different signal activation, cell surface markers and their functions. Based on the origin, tissue-resident macrophages can be divided into two subsets. One derives from the yolk sac and another population originates from hematopoietic progenitors and circulating monocytes. Bone macrophage includes bone marrow macrophage (erythroblastic island macrophage, hematopoietic stem cell niche macrophage), osteoclast and osteal macrophages (osteomacs).

M1 or classical activation of macrophages is an important inflammatory responser. Polarized M1 can produce high levels of reactive oxygen species (ROS), nitric oxide (NO), and pro-inflammatory cytokines such as interleukin IL-1, IL-2, IL-6, IL-12, TNF-α, and IFN-γ, which are involved in enhancing the host's defense response (Mosser, 2003; Genin et al., 2015). However, excessive stimulation of M1 macrophages can lead to tissue damage and autoimmune diseases (Mosser and Edwards, 2008). M2 macrophages mainly present in the subsiding phase of inflammation and are responsible for the production of anti-inflammatory cytokines and the clearance of apoptotic cells. Exposure to anti-inflammatory cytokines (IL-4, IL-10, and IL-13) or IL-1 receptor ligands or immune complexes and toll-like receptors (TLRs) can lead to M2 macrophage polarization (Mantovani et al., 2004; Guihard et al., 2012; Woo et al., 2015). M2 can produce anti-inflammatory cytokines such as chemokines ligands 18 (CCL-18), CCL-22, IL-10, and a small amount of IL-12 family members (Mosser and Edwards, 2008; Guo et al., 2019). In addition, M2 macrophages can produce a large number of osteogenic growth factors such as BMP-2 bone morphogenetic protein-2, a subclass of the TGF-β family and a potent promoter to osteogenic differentiation of MSCs (Champagne et al., 2002; Li et al., 2018), TGF-β (Assoian et al., 1987), osteopontin (Takahashi et al., 2004), and 1,25-dihydroxy-vitamin D3 (Kreutz et al., 1993). M2 can be further subdivided into M2a, M2c, and M2d by different signal activation, cell surface markers, and their functions (Figure 1) (Mosser and Edwards, 2008; Jetten et al., 2014; Murray et al., 2014; Roszer, 2015; Ogle et al., 2016; Arora et al., 2018).

Moreover, macrophages can also be divided into circulating and tissue-resident types. Tissue-resident macrophages are a diverse family of cells found in most organs (such as liver kupffer cells and alveolar macrophages in the lungs). Based on the origin, tissue-resident macrophages can be divided into two subsets. One derives from the yolk sac and another population originates from hematopoietic progenitors and circulating monocytes (Figure 1) (Heideveld and van den Akker, 2017). Tissue-resident macrophages express a large number of cell surface receptors, growth factors, proinflammatory and anti-inflammatory cytokines, and many other cell products (Jamalpoor et al., 2018). Most damage-related factors are first sensed by the resident macrophages, which become tense and recruit more macrophages when activated. Resident and recruited macrophages respond to their local environment and activate specific transcriptional programs that drive macrophages to a range of different phenotypes, from pro-inflammatory M1 macrophages to anti-inflammatory M2 macrophages (Xue et al., 2014; Juhas et al., 2018). Tissue damage caused by external (damage, chemicals, infection) and internal triggers (DNA damage, immune response) or by lack (nutrients, oxygen) and excess (sugar, cholesterol) factors may induce macrophage activation, sequentially lead to a disruption of the delicate balance between bone formation and resorption.

### One Subset of Bone-Resident Macrophages—Osteal Macrophages

Bone macrophage includes bone marrow macrophage (erythroblastic island macrophage, hematopoietic stem cell niche macrophage), osteoclast and osteal macrophages which also named osteomacs. Osteal macrophages were found in both periosteum and endosseous tissue, which support osteoblastic function and maintain bone homeostasis (Winkler et al., 2010; Cho et al., 2014b; Raggatt et al., 2014; Miron et al., 2016; Michalski and McCauley, 2017). Osteal macrophages account for about one sixth of the total cells in bone tissue (Chang et al., 2008), they present in resting bone tissue and increase in active bone anabolism sites. Osteal macrophages are a unique subset of bone-resident macrophages, close to the bone surface, f4/80 positive and trap-negative (Geissmann et al., 2010), adjacent to bone-forming cells (osteoblasts), dormant cells, and osteoclasts. The osteoblasts on the inner surface of cortical bone were mostly covered by f4/80^+^, CD68^+^, mac-3^+^, and trap-macrophages and be regulated by osteomacs (Chang et al., 2008; Batoon et al., 2019).

CSF-1 (colony stimulating factor 1) and various molecular markers are required for the proliferation and differentiation of mononuclear phagocyte progenitor cells to monocytes, osteal macrophages, and osteoclasts. Previous studies have shown that osteomacs on and in the periosteum are highly expressed with the mature macrophage marker CD169, which can be distinguished from osteoclasts (Mohsenzadegan et al., 2015; Batoon et al., 2019). So far, there is no unique marker between osteal cells and other bone macrophage subsets, but it is known osteal cells that in endosteum do not express ER-HR3 antigen which can be distinguished (Wu et al., 2016; Kaur et al., 2017).

## Communications Between Macrophages and Bone Cells

Bone is a kind of mineralized connective tissue, which plays the role of movement, support, and protection of soft tissues, storage of calcium and phosphorus, and preservation of bone marrow (Robling et al., 2006; Datta et al., 2008). Bone tissue is composed of roughly two parts: the dense layer and the spongy layer. The dense layer consists primarily of bone cells that make up the outermost layer of bone and functions primarily to support structural stability of the body and movement. The spongy layer is a trabecular, highly vascularized network of bone that houses the red and white marrow and is a hotbed of hematopoietic blood (Le et al., 2017). Despite bone is inert in appearance, it is a highly dynamic organ, constantly absorbed by osteoclasts and regenerated by osteoblasts, by which old bone is replaced by new bone. The equilibrium state of bone resorption and formation is regulated by local and systemic factors including cells, hormones, cytokines, etc. Here we focus on the relationship between bone derived macrophages and bone remodeling related cells.

### Macrophage and BMSCs

**Figure 2:**
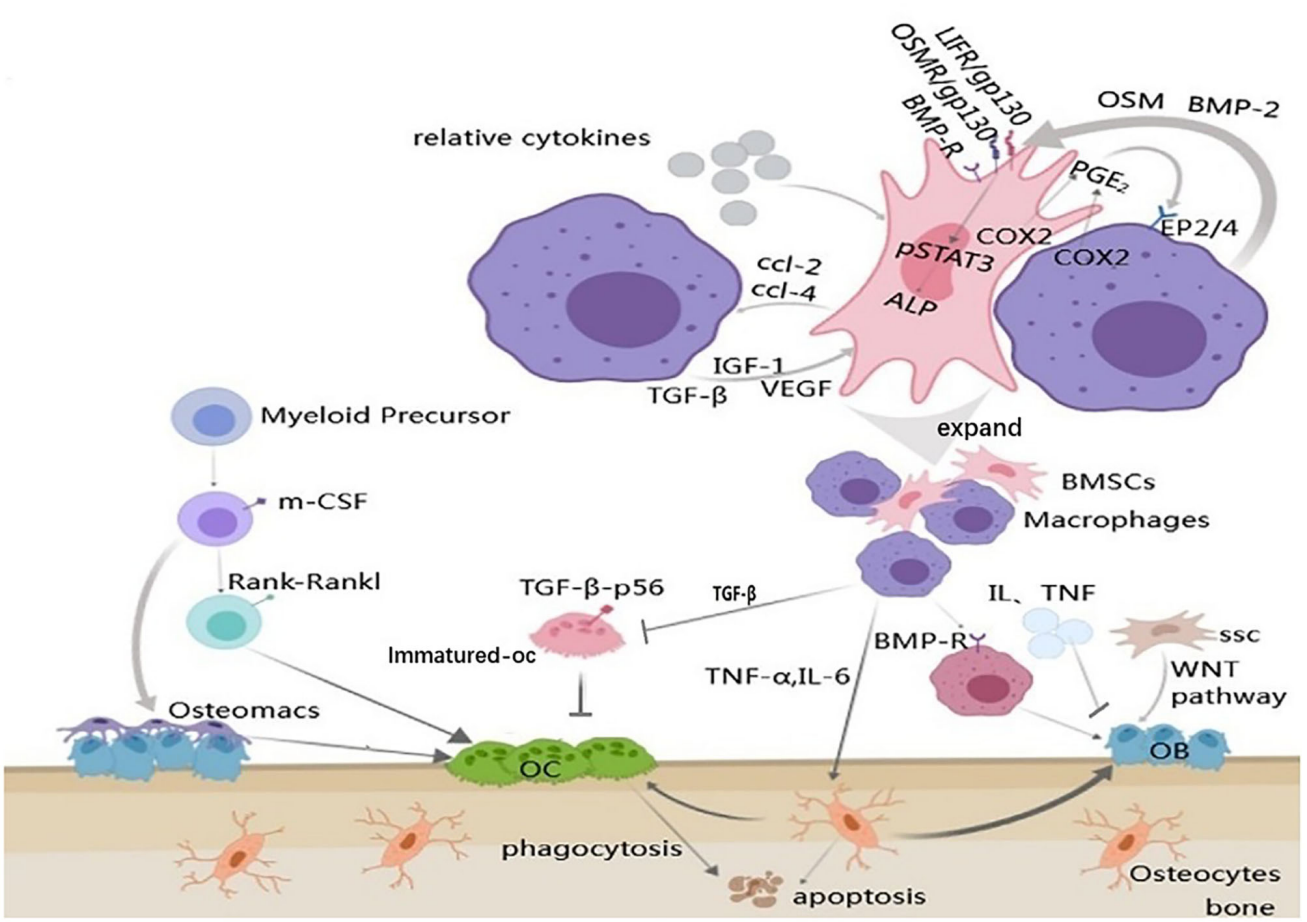
Communications between macrophages and bone remodeling cells. Arrow represents “stimulatory modification;” the gray triangle means “enlarged display;” the arrow with a line means “suppress.” Cell-cell communication between MSCs and macrophages results in the production of PGE2 and through the EP2/4 receptors to produce OSM. OSM acts on the MSCs via the gp130 receptors to activate STAT3 phosphorylation. Macrophage can also release BMP-2 to active the osteoblast differentiation genes, such as ALP, via the BMP-R in MSCs. MSCs can produce CCL-2 and CCL-4 to regulate macrophages and in reverse, this process is under the control of the macrophages; The differentiation of osteoblasts is regulated by variety of aspects including macrophages, osteocytes, inflammatory mediators and MSCs; Osteoclasts and osteomacs can derive from the same myeloid precursor, but it's unknown if the osteomac is a kind of the osteoclast precursors. Besides, macrophages mediate the growth of osteoclasts through various cytokines; Osteocytes apoptosis is under the regulation of macrophages.

Champagne is the first one to propose the mechanism by which macrophages contribute to osteogenic differentiation of bone marrow mesenchymal stem cells (BMSCs). In their study, the conditioned medium of the inactive J774A.1 mouse macrophage cell line was used for experiments, and it was found that the activity of alkaline phosphatase in human bone marrow mesenchymal stem cells was enhanced by the mediation of BMP-2 (Figure 2) (Champagne et al., 2002; Jamalpoor et al., 2018). A subsequent study co-cultured inactive human monocytes and human bone marrow mesenchymal stem cells (hbMSCs) and found that monocytes promoted MSC proliferation and increased expression of osteocalcin and osteopontin (Pirraco et al., 2013). Osteomacs regulate maintenance and proliferation of Nestin-positive MSC. These MSC express a variety of HSC retention factors and it is thought that macrophages talk to MSC via unknown secreted factors, excluding IL-1, IL-10, TNF-α, and insulin like growth factor 1 (IGF-1) (Heideveld and van den Akker, 2017). Nicolaidou et al. found that the number of macrophages in culture was positively correlated with bone formation when they co-culture human peripheral blood mononuclear cells (PBMCs) with hbMSCs (Nicolaidou et al., 2012). This process requires direct cell-cell contact to produce a soluble factor that induces STAT3 phosphorylation, known as oncostatin M (OSM). OSM is bound by two receptor complexes, consisting of a gp130 subunit and a leukemia suppressor receptor (LIFR). OSM gene deletion altered bone healing in the tibial injury model in mice. *In vitro* studies have described the stimulating effect of OSM produced by macrophages on the mineralization activity and differentiation of osteoblasts (Sims and Quinn, 2014). This process also depends on prostaglandin E2 (PGE2) and cox-2 (COX2) (Nicolaidou et al., 2012). Cell-cell contact between MSCs and macrophages produces PGE2 and induces OSM production through EP2/4 receptor on macrophages. Then OSM activates STAT3 phosphorylation through OSM and LIF receptors (Figure 2) (Nicolaidou et al., 2012; Horwood, 2016). Guihard also found the OSM signaling pathway. However, when hbMSCs were cultured in the conditioned medium of mononuclear cells treated with IL-4 or IL-10, there was no enhanced osteogenesis found and no OSM secretion detected (Guihard et al., 2012). Found in the study of inducing bone formation through bone Ti implants, the osteogenic differentiation of BMSCs could be changed by shifting the macrophage phenotype. Excessive polarization in the M1 direction leads to prolonged inflammation, while excessive polarization in the M2 direction leads to enhanced osteogenesis around Ti implants greatly (Wang et al., 2018). In contrast to the above studies, the effects of M0, M1, and M2 mouse bone marrow macrophages on osteogenic differentiation of mouse bone marrow mesenchymal stem cells have been studied. It was noted that all the macrophage subtypes could promote bone formation, and the M1-type macrophages had the greatest effect on bone formation (Lu et al., 2017). Studies have found that M1 macrophages increase the early and middle osteogenesis of MSCs, but do not increase matrix mineralization, while M2 macrophages co-culture can lead to increasing matrix mineralization. Moreover, it was found that the production of OSM increased in M1 culture and the production of BMP-2 increased in M2 co-culture, suggesting that different factors may be the driving factors for MSC differentiation in M1 and M2 cultures (Zhang et al., 2017).

In the co-culture model, MSCs significantly inhibited the production of LPS-induced pro-inflammatory cytokines (TNF-α, IL-1β, and IL-6) through iNOS and COX2-dependent pathways, and increased the secretion of IL-10 in macrophages by enhancing the production of PGE2 (Maggini et al., 2010). Experiments have proved that co-culture of MSCs and macrophages can significantly inhibit M1-macrophage polarization and induce M2 polarization (Kim and Hematti, 2009; Cho et al., 2014a). Human and mouse bone marrow mesenchymal stem cells secrete a large number of chemokines, including CCL-2 and CCL-4 (Seebach et al., 2014), which are the main chemokines for monocytes and macrophages (Mantovani et al., 2004). Combining INF-γ with another pro-inflammatory cytokine (TNF-β, IL-1β) activates bone marrow mesenchymal stem cells in damaged or inflammatory tissue, leading to increased secretion of various chemokines (Figure 2) (Ren et al., 2008). This MSC-mediated macrophage recruitment and macrophage phenotypic regulation may promote tissue regeneration (Bernardo and Fibbe, 2013).

### Macrophage and Osteoblasts

The purest form of bone formation is intramembrane ossification, in which the bone matrix is formed and deposited directly by osteoblasts. *In vivo*, osteal cells occur in multiple stages of intramembranous bone healing and form a unique canopy structure on mature osteoblasts. Osteal cells are in direct contact with stromal production and mineralized osteoblasts. Moreover, osteomacs promoted osteoblast differentiation, through the OSM-mediated tyrosine phosphorylation and interaction between the STAT3 and Yes-associated protein 1 (YAP1) (Wang et al., 2020). Depletion of osteomacs significantly inhibited the formation of new bone, however, specific enlargement of osteomacs resulted in a significant increase in new mineralized substrates (Raggatt et al., 2014). In MAFIA mice (a macrophage Fas-induced apoptosis mouse model), the bone transformation rate is significantly reduced (Cho et al., 2014b). TNF-α released by activated macrophages can stimulate osteoblast chemotactic effect *in vitro*, and can inhibit osteoblast differentiation in rheumatoid arthritis patients (Sun et al., 2018). IL-6 inhibits osteoblast differentiation and disrupts the balance of normal bone turnover (Harmer et al., 2018). Osteoblasts are derived from a subtype of skeleton stem cells (SSC) which belongs to bone marrow mesenchymal stem cells (Fierro et al., 2017). The WNT pathway is a major pathway for the transformation of SSC into osteoblasts. Macrophage-derived BMP-2 plays a critical role in inducing ossification by inducing alkaline phosphatase production through the signaling cascade of Wnt and Wnt/LRP5 in osteoblasts (Rawadi et al., 2003; Jamalpoor et al., 2018). Treatment of macrophages with BMP-2 antibody can prevent osteogenesis (Champagne et al., 2002). The conditioned medium collected from the BMP-2-stimulated macrophages also accelerated the osteogenic differentiation of the BMSC (Wei et al., 2018). In addition, BMP-2 affects the migration, recruitment and the differentiation of macrophages (Pardali et al., 2018). In addition to WNT, the macrophage derived-BMP can bind to the BMP receptor, which causes the dimerization of BMP-R and the phosphorylation of Smad proteins. Then, the phosphorylated molecule activates Runx2 to up-regulate OB activity and differentiation (Figure 2) (Kawabata et al., 1998). In a study of xenografts about deproteinized bovine bone matrix (DBBM), it was found that IL-10 released by macrophages can promote the osteogenic response of osteoblasts induced by macrophages (Shi et al., 2018).

### Macrophage and Osteoclasts

Osteal macrophages and osteoclasts are derived from myeloid progenitor cell precursors and can be stimulated by many of the same cytokines to function. But it must be emphasized that the osteomacs are not osteoclasts, because osteoclasts do not contain F4/80 Ag at all. Osteoclasts activated by pro-inflammatory stimuli may produce pre-osteoclast cytokines, including IL-6 and IL-1, which can promote the differentiation and/or function of osteoclasts. Therefore, osteoporosis may provide some candidate cellular mechanisms to explain why chronic inflammation and systemic infection often lead to osteopenia/osteoporosis (Chang et al., 2008). The generation of macrophage-derived osteoclasts can be activated by M-CSF and RANKL, and the blocking of the RANKL signaling pathway may prevent the progression of osteoporosis in mice (Jin et al., 2019). Macrophages can produce TGF-β1, which is essential for bone metabolism. NF-kB, composed of subunits such as p65, is a downstream transcription factor of the RANKL-RANKL signaling pathway (Park et al., 2017). Nuclear factor of activated T cells cytoplasmic 1 (NFATc1) is a master regulator of osteoclast differentiation (Okamoto and Takayanagi, 2011). Some studies have shown that TGF-β1 directly down-regulates NFATc1 activity by blocking the p65 in the receptor activator and then inhibits the generation and bone resorption of osteoclasts (Figure 2) (Tokunaga et al., 2020). Recently, the role of the NEMO protein (a core component of the NF-kB signaling pathway) in mouse bone marrow macrophages was investigated that if the lysine-270 (NEMO-Lys270) in NEMO protein was mutated to Ala, the NF-kB signal in bone marrow macrophages would lose control, leading to the accelerated production of osteoclasts (Adapala et al., 2020). What's more, studies have found that the overexpression of cr6 interaction factor-1 (Crif1) in mouse BMSCs can increase the secretion of RANKL through the cAMP/PKA pathway, and then combine with RANK on macrophages to promote the formation of osteoclasts *in vitro* (Xiang et al., 2020).

### Macrophage and Osteocytes

Osteocytes account for 90–95% of all bone cells and are the most abundant and long-lived cells (Franz-Odendaal et al., 2006). Activated macrophages can induce the production of pro-inflammatory cytokines such as IL-1, IL-6, and TNF-α, which may play a significant role in inflammatory bone loss. Activated fibroblast growth factor-23 secreted by osteocytes after activation induced by IL-1 and TNF-α may cause hypophosphatemia during sepsis. TNF-α attracts osteoclasts by inducing osteocyte apoptosis (Tan et al., 2006). IL-1 may cause the decrease of bone cell activity through the NF-kB/RANKL signaling pathway, and soluble IL-6 can increase osteocyte-mediated osteoclastic differentiation by activating JAK2 and RANKL during normal bone growth and bone remodeling (Wu et al., 2017; Yang and Yang, 2019). It was reported that purified osteocytes can express a much higher amount of RANKL, which bind with mouse bone marrow macrophage and support osteoclast differentiation (Elango et al., 2018). Inflammation is thought to be highly correlated with bone cell apoptosis. If macrophages in part of the femoral head are polarized to the M1 phenotype and up-regulate a large number of inflammatory mediators, they can promote bone cell apoptosis and accelerate femoral head necrosis (Figure 2) (Jin et al., 2020).

In summary, bone formation relies on the interaction of a variety of cells which reminded above and other cytokines including HIF-1α (Karshovska et al., 2020)and CSF-1 (Alexander et al., 2011; Raggatt et al., 2014)to maintain the dynamic balance of the skeletal environment.

## Macrophage-Derived Exosomes (Vesicles) Regulate Bone Metabolism

Intercellular communication is a key biological process that enables cells to coordinate their responses spatially and temporally to physiological changes. A fresh member of the intercellular communication system is the extracellular vesicle (EVs) (Théry, 2011) which is a small membrane derived phospholipid bilayer with a diameter between 30 and 2,000 nm. A vital class of EVs is exosomes (released by exocytosis, 30–150 nm diameter) (Colombo et al., 2014), has gained closely attention.

**Figure 3:**
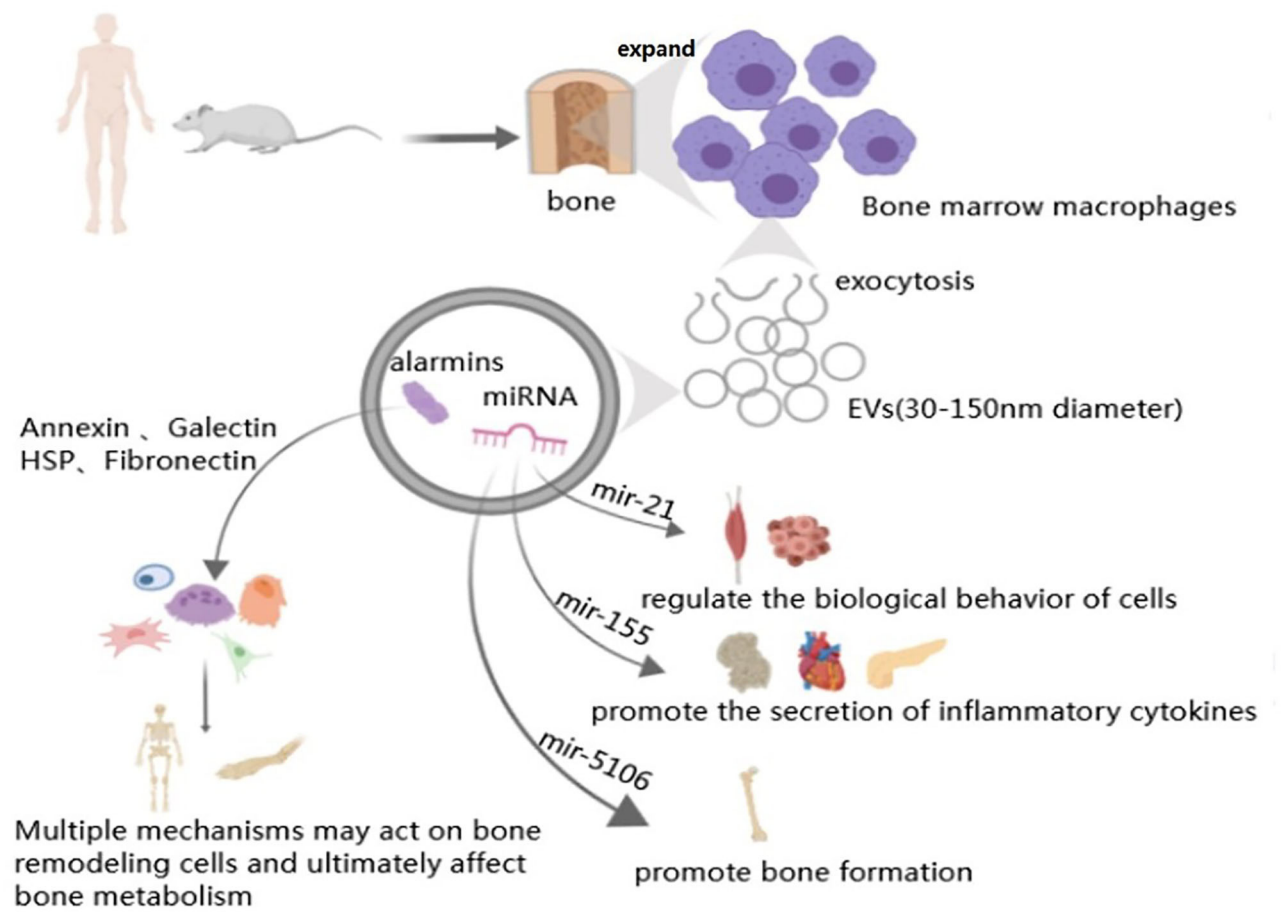
Macrophage-derived exosomes in bone metabolism. Arrow represents “stimulatory modification;” the gray triangle means “enlarged display.” The structure of macrophage derived exosomes is phospholipid bilayer with a diameter between 30 and 150 nm. Macrophage derived exosomes contain miRNAs (such as mir-21, mir-155, and mir-5106) and alarmins (such as Annexin, Galectin, HSP, and Fibronectin), all of them can through diverses signal pathways or release relative cytokines to participate in variety organs metabolism, including bone remodeling.

Recent studies have shown that macrophage-derived EVs play an important role in maintaining stability of the bone environment and bone remodeling (Table 1). In the study of BMP-2/macrophage-derived exosomes implantation of titanium nanotube, BMP2/macrophage-derived exosomes dramatically increased the expression of osteogenesis-related genes (ALP, osteopontin, Runx2, BMP-2, and BMP-7). Furthermore, exosome-encapsulated nanotubes activated autophagy of hBMSCs and altered the secretion of cytokines associated with bone remodeling. All of these indicate the pro-osteogenic role of the BMP-2/macrophage-derived exosomes (Wei et al., 2019). And studies have found that after tendon injury, the development of the fibrotic healing response impairs the function of the tendon and restricts the movement of the tendon. MicroRNAs are a same category of small, single-stranded non-coding RNAs discovered in diverse organisms, which can regulate the expression of mRNAs (Yang et al., 2017). Studies have found that exosome miR-21-5p secreted by bone marrow macrophages activates and promotes tendon cell fibrosis fiber formation by inhibiting the expression of Smad7 (Cui et al., 2019). After induction into M2 macrophages, murine bone marrow derived macrophages can secrete miR-21, interfere with the normal signal of the PTEN/PI3K/AKT signaling pathway, and thus regulate the biological behavior of a variety of tumors, including osteosarcoma (Figure 3) (Zheng et al., 2017; Yang et al., 2018). This finding serves as a potential target for the prevention and treatment of tendon adhesion and bone tumors. Bone marrow macrophage-derived miR-155 can be induced to release by the activators of pro-inflammatory M1 phenotype macrophages (LPS, IFN-α) and promote the secretion of inflammatory cytokines such as TNF-α, IL-12, thereby aggravating the inflammatory response in RA (Stanczyk et al., 2008), diabetes (Ying et al., 2017), and heart disease (Figure 3) (Heymans et al., 2013). The latest studies found that M2-type macrophages derived from mouse bone marrow macrophages enriched miR-5106. M2D-Exo containing miR-5106 can promote osteogenic differentiation of bone marrow mesenchymal stem cells and accelerate fracture healing *in vivo* by inhibiting the expression of osteogenic related genes SIK2 and SIK3 (Figure 3) (Xiong et al., 2020). Unexpectedly, compared to exosomes from M0 and M2, exosomes from M1 have a stronger stimulating effect on the proliferation, osteogenesis, and adipogenic differentiation of BMSCS and all three types of exosomes had an adverse effect on the chondrogenic differentiation of BMMSCs (Xia et al., 2020). The above findings indicate that the exact mechanisms of the macrophage-derived EVs still need to be further investigated and could be used as an effective therapeutic strategy for tissue regeneration.

**Table 1:** Macrophage-derived exosomes in bone metabolism.

**Cell of origin**	**Types**	**Mediators**	**Main biological effects**	**References**
Bone marrow macrophage	Exosomes	Undetected	Promote osteogenesis	Wei et al., 2019
	MicroRNAs	mir-21	Promote tendon cell formation and fibrosis by decreasing Smad7	Cui et al., 2019
			Interfere the PTEN/PI3K/AKT signaling pathway to regulate the biological behavior of cells	Zheng et al., 2017; Yang et al., 2018
		mir-155	Promote the secretion of inflammatory cytokines	Stanczyk et al., 2008; Heymans et al., 2013; Ying et al., 2017
		mir-5106	Inhibit the genes SIK2 and SIK3 to promote bone formation	Xiong et al., 2020
	Alarmins	Annexins	Induce bone resorption and help macrophages to work	Li et al., 2005; D'Souza et al., 2012; Stukes et al., 2016; McArthur et al., 2020; Xia et al., 2020; Xiong et al., 2020
		Galectins	Act on the differentiation of various bone cells	Andersen et al., 2003; Shimura et al., 2005; Nakajima et al., 2014; Weilner et al., 2016; Simon et al., 2017; Iacobini et al., 2018
		HSP; fibronectin	Functions through osteoblasts and osteoclasts.	Moursi et al., 1996; Koh et al., 2009; Gramoun et al., 2010; Notsu et al., 2016; Nakamura et al., 2019

Proteomic studies have shown that macrophage-derived EVs contain a large number of alarmins (one of endogenous molecules) (Figure 3). Annexins can stimulate bone resorption (Li et al., 2005), improve the phagocytic efficiency of macrophage (Stukes et al., 2016), which is related to Multiple myeloma (MM) (D'Souza et al., 2012) and play an auxiliary role of macrophages in infected tissues (Silva et al., 2019; McArthur et al., 2020; Sanches et al., 2020). Galectins mainly affect the differentiation of bone marrow stromal cells (Andersen et al., 2003), osteoblast, osteoclast (Shimura et al., 2005; Nakajima et al., 2014; Simon et al., 2017; Iacobini et al., 2018) and the osteogenesis of mesenchymal stem cells (Weilner et al., 2016). What's more, HSP-60 (Koh et al., 2009), HSP-70 (Notsu et al., 2016; Nakamura et al., 2019) and fibronectin are also primarily concerned with osteoblasts (Moursi et al., 1996) and osteoclasts (Gramoun et al., 2010) to come into play (Table 1).

Macrophages play a critical role in bone remodeling as a source of vehicle-carried alarmins. In conclusion, future studies should be more detailed to determine the contribution of the macrophage-derived exosomes.

**Figure 4:**
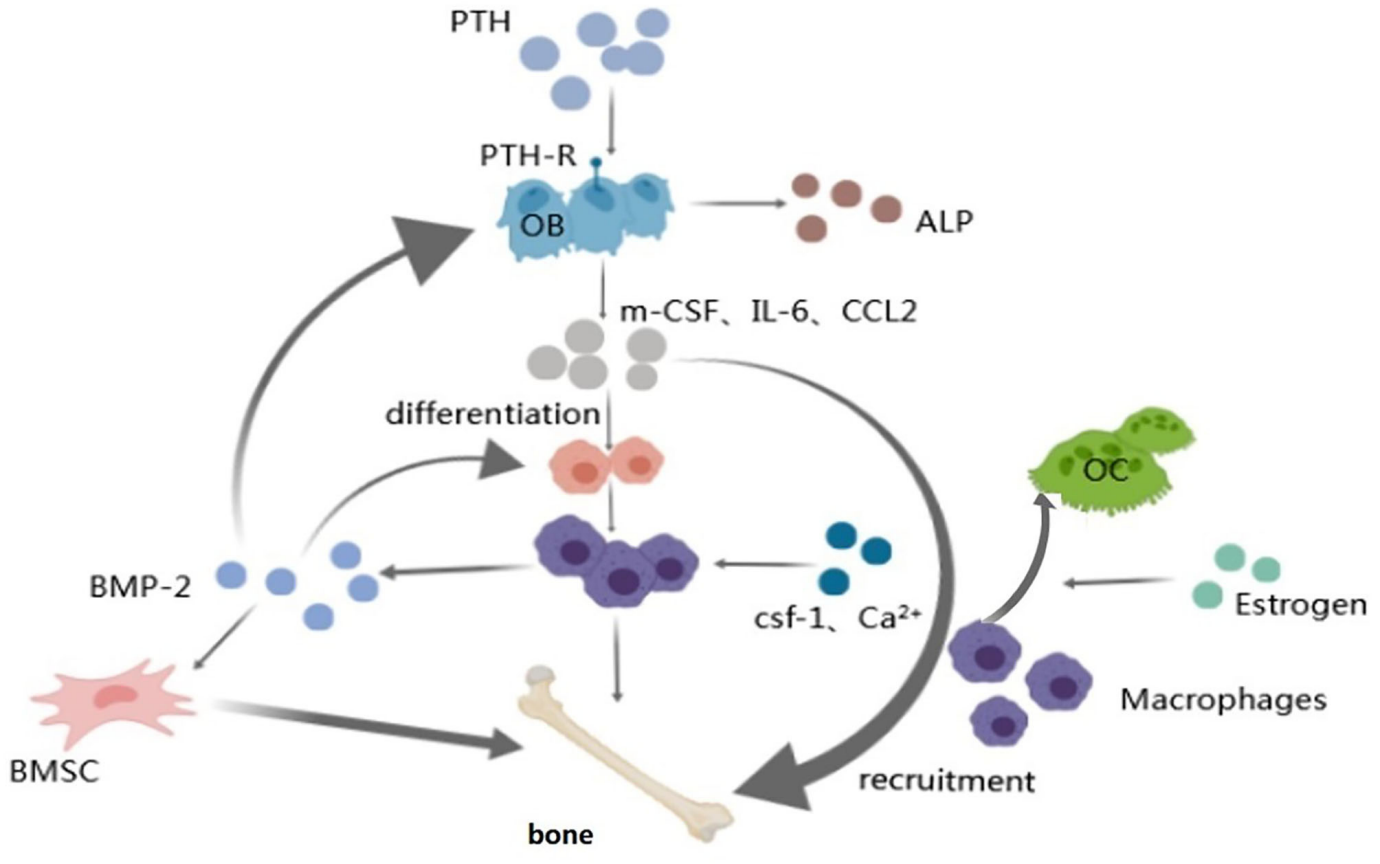
Macrophage and regulatory hormones and factors. Arrow represents “stimulatory modification;” the gray triangle means “enlarged display.” Macrophages play a part in the anabolic response to intermittent PTH therapy via osteoblasts which can secrete factors (IL-6, etc.) that promote the differentiation and recruitment of macrophages; Worthy to be mentioned the work of CSF-1 and Ca^2+^ on macrophages; Macrophage-derived BMP-2 has effect on BMSCs, osteoblasts and macrophages via BMP-R to accelerate the osteogenic differentiation so as to make bone metabolism balance; Estrogen regulates bone homeostasis mainly through osteoblasts and osteoclasts. Estrogen mainly affects the activity of macrophages by interfering with the macrophage-mediated osteoclast formation process.

The body's regulatory agents are complex and poorly understood, and future efforts would be made to unravel them (Figure 4).

## Macrophage and Bone Remodeling-Related Bone Diseases

Tissue homeostasis requires a tight tissue system of various cell types to maintain. If the balance is disrupted, the progression of abnormal disease states may continue. Macrophages are involved in the development of many diseases with various phenotypes, but the direct link between macrophage function and bone-related diseases has not been thoroughly studied.

### Osteoporosis

Osteoporosis is a common disease characterized by the destruction of bone mass and microstructure, resulting in brittle fractures. The characterize of age-related osteoporosis is reduced bone formation and increased marrow fat accumulation (Li et al., 2015). People with osteoporosis are under a 40% lifetime risk of fractures, most often in the spine, hip or wrist. As the aging of the population, postmenopausal osteoporosis, in particular, will further increase (Rachner et al., 2011). In addition, the 12-month mortality rate for osteoporotic hip and spinal fractures is up to 20%, and the incidence of fracture complications such as pneumonia or thromboembolic disease increases due to chronic fixation (Center et al., 1999).

**Figure 5:**
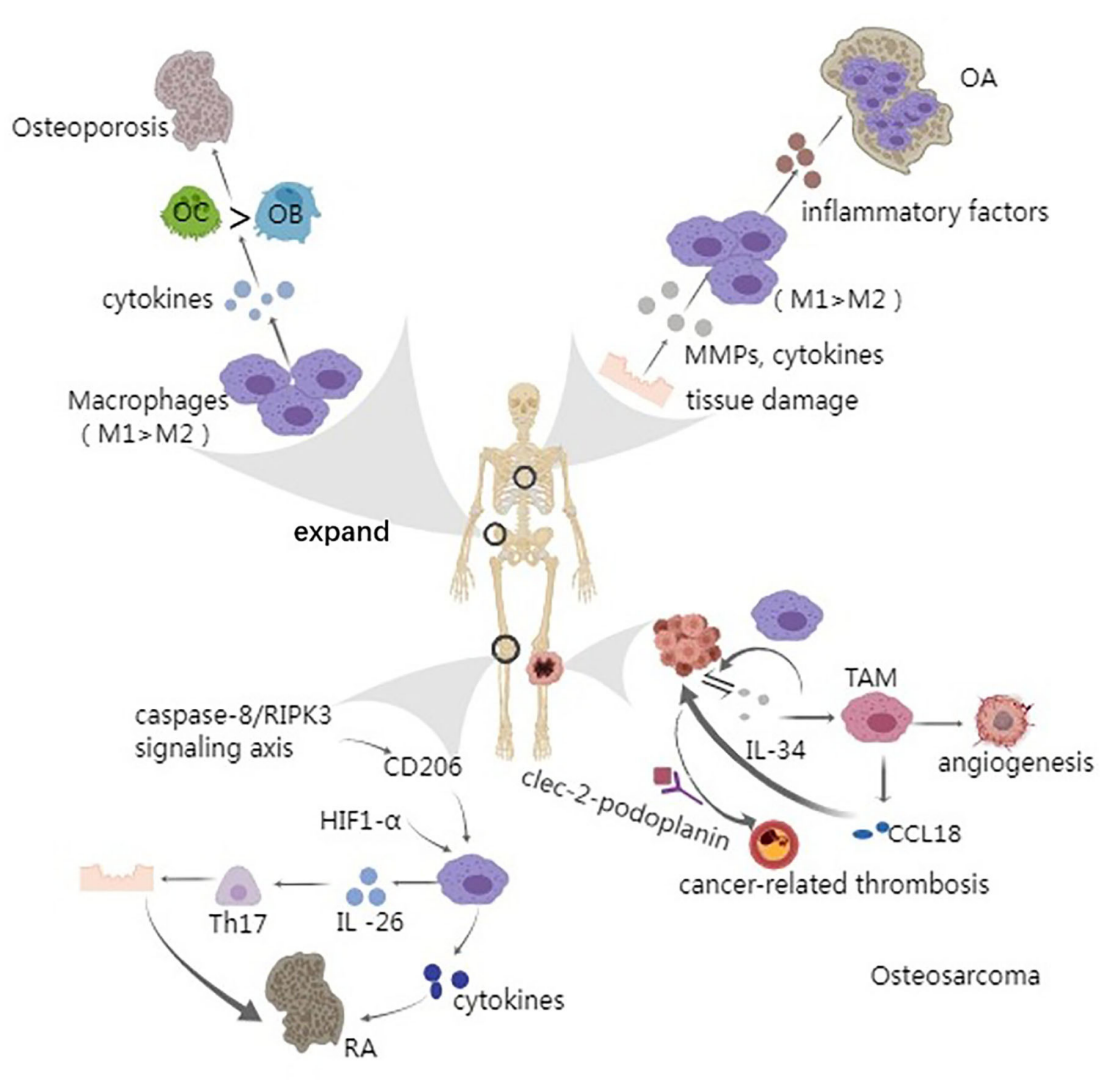
Macrophage and bone remodeling related diseases. Arrow represents “stimulatory modification;” the gray triangle means “enlarged display.” Osteoporosis is an age-related bone disease to some extent. The release and accumulation of proinflammatory cytokines can active osteoclasts and thus greatly accelerate the process of osteoporosis; The patients of osteoarthritis have a vital inducer that is tissue damage which can release various cytokines, leading to the imbalance between M1 and M2 macrophages. Usually, the quantity of M1 prevails, leading to the aggravation of OA; Caspase-8/RIPK3 signaling pathway and HIF1-α have been found to play a role in regulating macrophages, through CD206 and glycolysis, respectively. Besides, macrophages can secret IL-26, an inducer of Th17 cells which can cause autoimmune tissue damage; Osteosarcoma always occurs in the metaphyseal region of the long bone and the growth and metastasis of tumor cells have a closely relationship with IL-34 and CCL18. Moreover, tumor cell-derived IL-34 stimulates the angiogenesis of TAM. Cancer-related thrombosis could be made by the interaction of clec-2-podoplanin and the ectopic expression of podoplanin in patient's macrophages is the inducer, leading to the severe complications.

Aging is usually accompanied by an irreversible recession in physiological functions. Age-associated metabolic dysfunction includes, but is not limited to, increased fat mass accumulation and insulin sensitivity deterioration (Huang et al., 2020). Aging is usually accompanied by a decrease in M2-type macrophages and an increase in proinflammatory factors, which promote the M1 transformation of the macrophage phenotype to aggravate the symptom. A study of transplanted bone marrow from 4-week-old mice into 12-month-old mice found improved fracture healing in older mice, which was attributed to younger inflammatory cells participating in the repair process (Xing et al., 2010). Cytokines secreted by macrophages related to osteoclasts including IL-1, IL-6, IL-18, IL-23, IL-27, and TNF-α may promote osteoclast differentiation and activation in an inflammatory state (Figure 5) (Yang and Yang, 2019). As mentioned, macrophages regulate osteoclasts and osteoblasts mainly through inflammatory cytokines to mediate the occurrence of osteoporosis.

In addition to inflammatory response, latest studies have identified new regulatory mechanisms of macrophages in osteoporosis. They employed mice BMMs modified for over expression or inhibition of miR-128 levels to determine its effect on osteoclast differentiation. *In vitro* experiments showed that miR-128 may play a regulatory role in BMMs through miR-128/SIRT1/NF-kB signaling axis, and over expression or inhibition of miR-128 can significantly increase or decrease the occurrence of macrophage-derived osteoclasts, respectively (Shen et al., 2020). Further studies found that miR-506-3p can selectively inhibit NFATc1 in RANKL-induced activated rat BMMs, further reduce the release of bone resorption enzymes, thus relieving osteolysis (Dinesh et al., 2020). What's more, both the smo-GLI1/2 axis (Hh ligands permitting the activation of Hh signal transducer smoothened (SMO) and transmitting intracellular signaling through transcription factors of the GLI family)in the Hedgehog (Hh) signaling pathway (Kohara et al., 2020) and delphinidin (Imangali et al., 2020) can mediate the regulation of osteoclasts by macrophages.

The new studies provide an important basis for the treatment of senile osteoporosis by preventing osteoclast formation which derived from macrophages, but the exact mechanism of macrophages in osteoporosis has yet to be investigated.

### Osteoarthritis

Osteoarthritis (Murray et al., 2014), the most common form of joint disease, is characterized by cartilage destruction, synovial fibrosis, osteophyte (osteophyte at the edge of the joint) formation and pain, and sometimes swelling of certain joints such as the knee, hip, hand, and spine (Hügle and Geurts, 2017). Depending on the world health organization (WHO), the prevalence of OA worldwide exceeds 150 million (Harrell et al., 2019). The exact mechanism of OA appears to be the result of complex interactions among mechanical, cellular, and immune factors. Synovitis has turned out to be found in the early and late stages of most patients with OA (Sellam and Berenbaum, 2010), and the accumulation of macrophages in the synovium is a feature of synovitis (Sun et al., 2016).

The high proportion of M2 macrophages in the synovial tissues has certain clinical diagnostic significance for OA (Chen et al., 2020), and the pro-inflammatory M1 macrophages are also increased significantly (Sun et al., 2017). Some studies have confirmed that macrophages play an important role in the occurrence of OA through inflammatory factors, cytokines and proteins, whether it is inflammatory or mechanical injury (Bondeson et al., 2010). Since M1 macrophages are considered to be pro-inflammatory while M2 macrophages are anti-inflammatory, the degree of imbalance between M1 and M2 macrophages in OA is related to the severity of OA (Xue et al., 2019). In OA, potential mediators (MMPs, cytokines, and growth factors due to cartilage injury) leak into synovial fluid through damaged cartilage and activate synovial macrophages. Subsequently, the proinflammatory cytokines, catabolism, and anabolic factors are released, which induce osteophyte formation (Figure 5) (Liu-Bryan, 2013). OA was induced by intra articular injection of collagenase (collagenase-induced osteoarthritis (CIOA) and surgical medial meniscus instability (DMM) and the result showed that compared with M2 macrophages, M1 polarization of synovial macrophages was increased in the CIOA and DMM circu;amodels (Zhang et al., 2018). All above studies indicate that M1 macrophages are potential therapeutic targets for OA therapy.

### Rheumatoid Arthritis

Rheumatoid arthritis (RA) is a chronic immune-mediated inflammatory disease that affects nearly 1% of the world's population. It mainly affects synovial tissue, cartilage, and bone, leading to delayed joint damage, pain, swelling, and stiffness (McInnes and Schett, 2011). RA patients are at a higher risk of serious infection, respiratory disease, osteoporosis, cardiovascular disease, cancer, and death than the general population.

The exact pathogenesis of RA is unknown, but the activated macrophages play an important role in the pathogenesis. Caspase-8 is a promoter of apoptosis and an inhibitor of cell necrosis. Studies have found that the caspase-8/RIPK3 signaling axis is involved in maintaining stability of macrophages in synovial tissues, thereby limiting arthritis. The lack of caspase-8 in mouse synovium macrophage reduces the expression of CD206, resulting the inadequate of caspase-8-deficient macrophages to endocytosis of cellular debris caused by joint inflammation, thereby failing to control subsequent inflammation (Figure 5) (Dominguez et al., 2017). IL-34 is a pleiotropic cytokine and it's expression is associated with inflammatory diseases involving excessive proliferation of monocytes/macrophages (Ushach and Zlotnik, 2016). The plasma concentration of IL-34 in synovial fluid and serum is low (Nandi et al., 2012; Tian et al., 2013), but in rheumatoid arthritis, the content of IL-34 is dramatically increased, hence it's level is a useful biomarker for predicting the progression of rheumatoid arthritis (Ding et al., 2015; Liu et al., 2018; Ge et al., 2019). IL-34 and CSF-1R act in combination on mononuclear cell line (THP-1) can through a series of reactions to promote the production of helper T (Th17) cells, which is the main driver of autoimmune tissue damage, especially RA (Kuwabara et al., 2017). Additional data indicated that mononuclear/macrophage-derived IL−26 stimulation is also an important inducer of Th17 cells in RA (Figure 5) (Kragstrup et al., 2018). In addition, scientists have found that increasing the amount of anti-inflammatory macrophages by decreasing FLIP (flice-like inhibitory protein, highly expressed in RA synovial macrophages) in macrophages may be an effective treatment for suppressing inflammation (Huang et al., 2017). What's more, it is now becoming clear that metabolic pathways are the characteristic of rheumatoid arthritis and they may be the potential therapeutic targets (Sanchez-Lopez et al., 2019). Glycolysis is the preferred source of ATP under anoxic conditions (Epstein et al., 2017). Inflammatory joints are usually in a severe hypoxia environment, which results in the synovial fluid hypoxia inducing factor 1 α (HIF1-α) generation increases. The HIF1-α can inhibit the progression of glycolysis to stimulate the production of IL-1 and other pro-inflammatory cytokines by macrophages (Figure 5) (Tannahill et al., 2013), thus to accelerate the incidence of RA, increase oxidative damage and cartilage erosion (Hua and Dias, 2016).

### Osteosarcoma

Osteosarcoma is the most common primary malignant bone tumor. It can occur in any bone but most seen in the metaphyseal region of the long bone, and initially presents as progressive pain and/or swelling. Risk factors are various (Harrison and Schwartz, 2017) and this tumor tend to involve the lung (Kelleher and O'Sullivan, 2017).

Both cancer cells and immune cells can secrete various interleukins such as IL-34 (Franzè et al., 2020), which can increase the recruitment of M2 macrophages and promote the growth and metastasis of osteosarcoma (Ségaliny et al., 2015). There is increasing evidences that IL-34 promotes tumorigenesis through autocrine and paracrine mechanisms. In the autocrine pathway, IL-34 interacts with the M-CSF receptor on cancer cells to activate signaling pathways, stimulate the growth, and spread of cancer cells and increase their resistance to chemotherapy drugs (Figure 5) (Baghdadi et al., 2016). In the paracrine pathway, IL-34 produced by tumor cells and/or immune cells triggers M-CSF1-R signaling in tumor-associated macrophages (TAMs), thereby promoting recruitment of TAM to the tumor area, facilitating the formation of new blood vessels and the exosmosis of immunosuppressive cells (Figure 5) (Mantovani et al., 2017). CCL-18 (c-c motif chemokine ligand 18) is an element secreted by TAM. Studies have shown that CCL-18 can be the activator of the EP300/UCA1/Wnt/hy-catenin pathway and promote the proliferation and migration of OS cells (Figure 5) (Su et al., 2019). Patients with cancer have an increased risk of thromboembolism. Platelet-activated receptor c-type lectin-like receptor 2 (clec-2) is almost specifically expressed in human platelets/megakaryocytes. The endogenous ligand podoplanin is a membrane protein. Patients with osteosarcoma may have ectopic expression of podoplanin in macrophages, and the interaction of clec-2-podoplanin stimulates the formation of cancer-related thrombosis, thus aggravating the patient's condition (Figure 5) (Suzuki-Inoue, 2019). The exact mechanism of osteosarcoma is still unknown, but recent studies on macrophages may provide new methods for clinical treatment and prevention of complications in patients with osteosarcoma.

Bisphosphonates, PTH, estrogen, or selective estrogen receptor modulators (SERMs) and calcitonin have been used to treat osteoporosis. Non-steroidal anti-inflammatory drugs are commonly used in rheumatoid. Moreover, surgical treatment is considered the first choice for the treatment of OA and osteosarcoma. But long-term use of PTH/bisphosphonates can increase the risk of severe side effects (osteosarcoma, mandibular necrosis, and atypical femoral fractures). Surgical treatment is unsuitable for the patients who cannot tolerate it. Thus, it seems that new treatments for common bone diseases are especially important in terms of conservative treatment. At present, we have known that bone tissue macrophages can communicate with a variety of cells in the bone by secreting of a series of hormones, cytokines, and exosomes. They mediate bone remodeling in various aspects and provides a new direction for clinical treatments.

## Conclusions and Future Directions

Current knowledge of macrophages has been outlined, including their polarization, support for bone formation, potential role in bone biology, and regulation of bone metastasis. Osteal macrophages construct the cell canopy structure at the bone reconstruction site, anabolic cytokines promote bone formation and coordinate the coupling between osteoclasts and osteoblasts. However, the study of osteal macrophages is a relatively new research field, and many important and puzzling research problems still exist. The identification of specific markers of osteal macrophages remains to be explored, and the mice with specific Cre expression in BMMs/osteal macrophages is also expected to provide greater convenience for scientific research.

The potential role of microRNAs and protein profiles in exosomes, secreted by bone-resident macrophages, remains the critical issues. The prospect of improving patient outcomes and designing new therapeutic approaches are the eventual goals by better understanding the change of macrophages in the state of aging and figuring out the effects of the change on bone remodeling related diseases. Presently, new treatments targeting macrophages mainly focus on mediating the inflammatory response while lack the specific methods targeting the communications of bone-resident macrophages and other cells. Thus, validation of the role of macrophages in human bone disease requires further investigation. Targeting bone-resident macrophages may be a powerful tool for the treatment of bone remodeling related diseases.

## Author Contributions

CL conceived the presented idea and supervised the written of this work. KC wrote the manuscript with supports from CL, XL, MH, YJ, CH, WH, JH, LL, and MY. All authors discussed the results and contributed to the final manuscript.

## Conflict of Interest

The authors declare that the research was conducted in the absence of any commercial or financial relationships that could be construed as a potential conflict of interest.
